# Eosinophilic Folliculitis Presenting as Persistent Pruritic Facial Papules: A Diagnostic Challenge

**DOI:** 10.7759/cureus.106653

**Published:** 2026-04-08

**Authors:** Jia Qi Adam Bai, Muhammad N Mahmood, Eunice Y Chow

**Affiliations:** 1 Faculty of Medicine, University of Ottawa, Ottawa, CAN; 2 Department of Laboratory Medicine and Pathology, University of Alberta, Edmonton, CAN; 3 Division of Dermatology, University of Alberta, Edmonton, CAN

**Keywords:** case report, eosinophilic folliculitis, facial papules, pruritic dermatoses, systemic lupus erythematosus

## Abstract

Eosinophilic folliculitis is a rare inflammatory dermatosis characterized by recurrent pruritic follicular papules or pustules with eosinophil-predominant inflammation. Although classically associated with immunosuppression, it demonstrates a broad clinical spectrum and may present diagnostic challenges. We report a 50-year-old woman with a several-year history of recurrent, severely pruritic facial papules initially attributed to chronic spontaneous urticaria, mastocytosis, or an atypical presentation of papulopustular rosacea. Histopathologic interpretation was initially reported as being consistent with an arthropod bite reaction. However, the failure to respond to all directed therapies prompted clinical suspicion of eosinophilic folliculitis. Targeted dermatopathologic re-review revealed a folliculocentric eosinophilic infiltrate diagnostic of eosinophilic folliculitis. The patient demonstrated a complete and reproducible response to oral indomethacin. Over longitudinal follow-up, she was subsequently diagnosed with systemic lupus erythematosus more than five years after the onset of the cutaneous symptoms. This case highlights the variable clinical and histopathologic presentation of eosinophilic folliculitis, the importance of clinicopathologic correlation in refractory facial eruptions, and the potential association with underlying immune dysregulation.

## Introduction

Eosinophilic folliculitis is a rare inflammatory dermatosis characterized by recurrent pruritic follicular papules or pustules with eosinophil-predominant inflammation [1,2]. Eosinophilic folliculitis comprises several clinical subtypes, including classic (i.e., Ofuji disease), immunosuppression-associated forms most commonly seen in patients with human immunodeficiency virus infection, and less common infantile variants, each with overlapping but distinct clinical features [3,4]. Although eosinophilic folliculitis typically presents with intensely pruritic follicular papules and pustules distributed on the face, scalp, and upper trunk, significant clinical heterogeneity has been described, including urticarial or papular morphologies without overt pustulation, which may obscure the diagnosis [5].

The pathogenesis of eosinophilic folliculitis remains incompletely understood but is thought to involve eosinophil-rich inflammation driven by immune dysregulation, including T helper 2-skewed cytokine signaling, sebaceous antigens, and prostaglandin-mediated pathways [3,4]. Although eosinophilic folliculitis has most commonly been described in idiopathic settings or in association with immunosuppression, rare cases have been reported in association with systemic lupus erythematosus [6]. Given its variable clinical presentation and overlapping features with more common dermatoses such as rosacea, arthropod reactions, and chronic urticaria, eosinophilic folliculitis is frequently misdiagnosed, and histopathologic findings may be subtle or heterogeneous, particularly when biopsies are obtained from non-follicular sites.

We report a case of facial eosinophilic folliculitis that was initially attributed to clinical differential diagnoses of chronic urticaria, mastocytosis, or rosacea. It was only after failure of all treatments directed at these conditions that eosinophilic folliculitis was considered clinically, prompting targeted dermatopathologic re-review of the existing biopsy, which then confirmed the diagnosis. The patient achieved complete resolution with indomethacin and was subsequently diagnosed with systemic lupus erythematosus more than five years after the onset of cutaneous symptoms, raising the possibility of an underlying immunologic association.

## Case presentation

**Figure 1:**
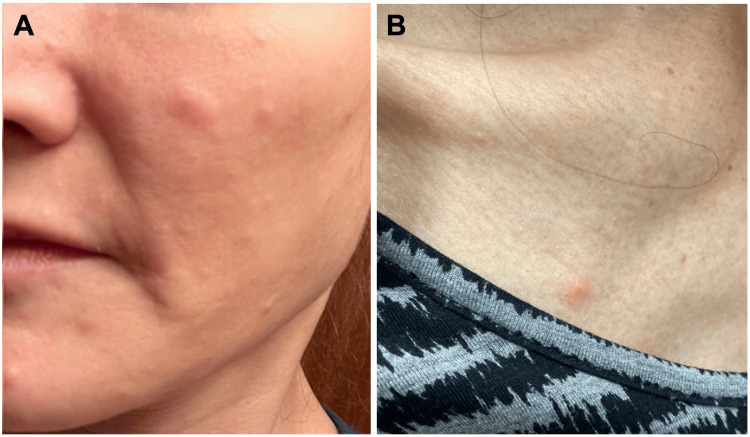
Clinical presentation of eosinophilic folliculitis. (A) Representative erythematous, edematous papules on the cheek without pustulation. (B) Concurrent erythematous papule on the right upper chest, consistent with extrafacial involvement.

A 50-year-old woman was referred by her family physician for *facial hives* to a tertiary care academic dermatology clinic in Edmonton, Alberta. She presented with a several-year history of recurrent, severely pruritic papules affecting the cheeks, forehead, chin, upper chest, and neck (Figures 1A-1B). She had a 10-year history of positive anti-nuclear antibody with negative extractable nuclear antibody screens and a persistently low white blood cell count ranging from 2.6 to 3.8 x 10^9^/L (reference range: 4.0-11.0 x 10^9^/L). She had no other signs or symptoms of systemic lupus erythematosus at that time. Individual lesions were erythematous and edematous, measured approximately 5-8 mm, and often persisted for several days. The eruption was socially distressing and refractory to multiple therapies, including oral antihistamines, topical corticosteroids, topical calcineurin inhibitors, topical anti-acne treatments, topical ivermectin, oral doxycycline, and oral prednisone. Given the facial-predominant distribution and urticarial morphology, initial clinical considerations included chronic spontaneous urticaria despite the atypical persistence of individual lesions, as well as arthropod bite reactions, papulopustular rosacea, and mastocytosis. A trial of omalizumab was initiated for presumed chronic spontaneous urticaria, resulting in only partial and inconsistent improvement after six months of therapy.

**Figure 2:**
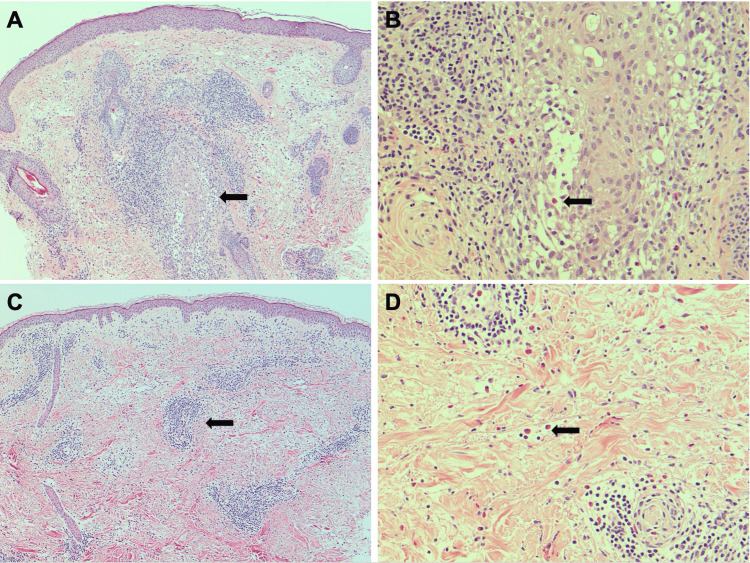
Histopathological evaluation of skin biopsies. (A) Low-power examination of the right cheek punch biopsy shows folliculocentric pattern of inflammation (hematoxylin and eosin stain, original magnification x50, arrow marks hair follicle). (B) High-power examination of the right cheek punch biopsy displays perifollicular and intrafollicular predominantly lymphocytic infiltrate with easily recognizable eosinophils (hematoxylin and eosin stain, original magnification x200, arrow marks eosinophil). (C) Low-power examination of the right upper chest discloses superficial and deep perivascular lymphocytic infiltrate (hematoxylin and eosin stain, original magnification x50, arrow marks perivascular infiltrate). (D) High-power examination of the right upper chest displays many perivascular and interstitial eosinophils in the reticular dermis (hematoxylin and eosin stain, original magnification x200, arrow marks eosinophils).

Punch biopsies were obtained from a facial lesion and a concurrent papule on the upper chest. Initial histopathologic interpretation described findings consistent with an arthropod bite reaction. However, this diagnosis was inconsistent with the chronic, recurrent nature of the eruptions, as both facial and chest lesions recurred across all seasons over several years, with the facial lesions being of greater concern to the patient due to their visible location. After failure of all directed therapies, eosinophilic folliculitis was considered clinically, and the existing biopsies were resubmitted for dermatopathologic re-review with this clinical suspicion. On re-review, two separate patterns of inflammation were identified in the biopsies. The facial biopsy demonstrated a folliculocentric pattern of inflammation with perifollicular and intrafollicular predominantly lymphocytic infiltrate with easily recognizable eosinophils (Figures 2A-2B). Neutrophilic pustules were not identified, periodic acid-Schiff stain was negative for fungi, and follicular mucinosis was not observed. These findings were reinterpreted as consistent with eosinophilic folliculitis. The upper chest biopsy demonstrated a different pattern characterized by superficial and deep perivascular lymphocytic infiltrate (Figure 2C). Follicular involvement was not seen; however, many perivascular and interstitial eosinophils were noted in the reticular dermis (Figure 2D). Although the microscopic changes on chest biopsy could be interpreted as an arthropod bite reaction, perifollicular eosinophilic infiltration is more commonly seen in facial eosinophilic folliculitis, whereas a perivascular pattern is more frequently observed in extrafacial eosinophilic folliculitis [7]. In this clinical context, the chest biopsy was reinterpreted as a manifestation of extrafacial eosinophilic folliculitis. Notably, peripheral eosinophil counts remained within normal limits throughout the clinical course, despite histopathologic evidence of eosinophil-predominant inflammation.

**Figure 3:**
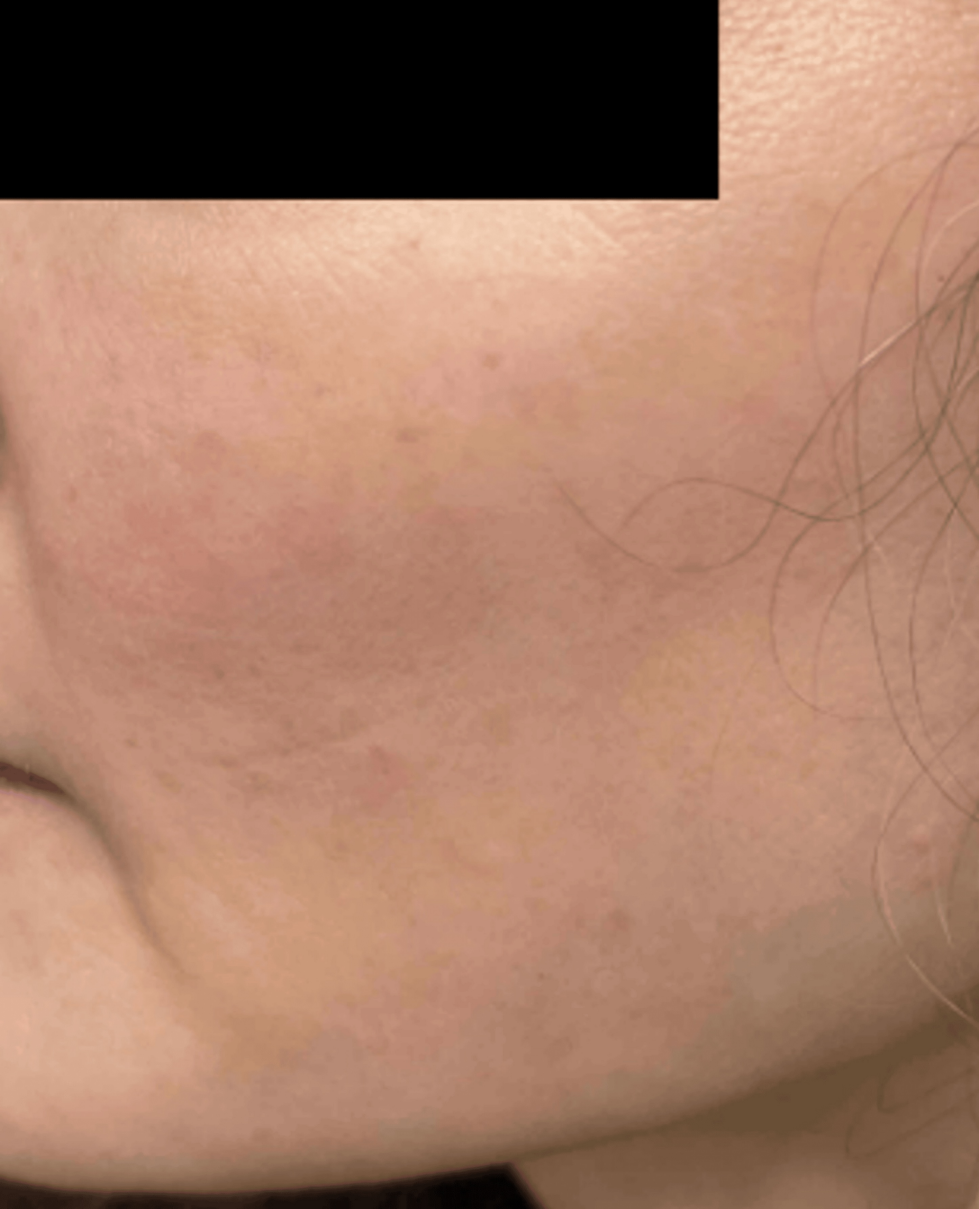
Clinical image after treatment response. Complete resolution of the papules, four weeks after initiation of indomethacin.

Based on clinicopathologic correlation, treatment with oral indomethacin 25 mg twice daily was initiated. Within approximately four weeks, the patient experienced complete resolution of her eruption (Figure 3). After discontinuation of indomethacin, lesions recurred approximately one year later, with complete clearance again achieved following reintroduction of therapy, confirming a reproducible treatment response. Over subsequent follow-up, she required only intermittent indomethacin for occasional flares, with sustained disease control.

During longitudinal follow-up, the patient developed severe fatigue and generalized arthralgias and was also diagnosed with systemic lupus erythematosus with elevated anti-double stranded DNA antibodies and low complement levels, raising the possibility of an underlying immune dysregulatory association.

## Discussion

Eosinophilic folliculitis has most commonly been described in idiopathic settings or in association with immunosuppression, particularly human immunodeficiency virus infection [4,8]. However, emerging reports suggest that it may rarely occur in the context of autoimmune disease [6]. Notably, her eosinophilic folliculitis preceded the diagnosis of systemic lupus erythematosus by more than five years. Although eosinophilic folliculitis is most commonly associated with human immunodeficiency virus-related immunosuppression or idiopathic disease, rare cases in association with autoimmune disorders have been reported [6]. The temporal relationship in this patient raises the possibility that eosinophilic folliculitis represents an early cutaneous manifestation of immune dysregulation rather than a coincidental finding.

The clinical presentation of eosinophilic folliculitis is considerably more variable than its classic description suggests, and this variability is a major contributor to diagnostic delay. The prototypical eruption of classic eosinophilic folliculitis often consists of intensely pruritic follicular papulopustules arranged in arcuate or annular plaques with central clearing and peripheral extension, predominantly involving the face and seborrheic areas [9]. However, significant morphologic heterogeneity exists across subtypes. In the immunosuppression-associated form, the annular configuration and facial predilection are frequently absent, with lesions instead presenting as scattered perifollicular erythematous papules distributed across the head, neck, and upper trunk. Furthermore, facial and extrafacial lesions have been shown to differ not only in distribution but also in clinical morphology, with annular plaques being significantly more common in facial disease, whereas extrafacial involvement more frequently presents as isolated papules without plaque formation and is more commonly associated with underlying immunosuppression, as observed in this patient’s chest lesions [7]. Notably, a variant of classic eosinophilic folliculitis termed episodic eosinophilic dermatosis of the face has been described in which pustule formation and peripheral extension are entirely absent, yet the condition retains its characteristic response to indomethacin [1]. Collectively, these observations underscore that the absence of pustules, annular plaques, or classic facial morphology does not exclude eosinophilic folliculitis from the differential diagnosis of a refractory pruritic eruption.

The histopathologic variability of eosinophilic folliculitis is well documented in the literature. A detailed review of 52 biopsies demonstrated that spongiosis of the follicular epithelium was present in all cases and that the isthmus was the dominant level of inflammatory involvement [10]. However, frank intrafollicular eosinophilic pustules were identified in only a minority of specimens. Perifollicular lymphocytic infiltrates with relatively few eosinophils were the most consistent finding, and microorganisms were notably absent from areas of inflammation in all study biopsies. This histopathologic breadth has direct clinical relevance, as no single biopsy feature is pathognomonic and the diagnosis therefore relies on clinicopathologic synthesis rather than histology alone [9]. A proposed diagnostic algorithm recommends suspecting eosinophilic folliculitis in any patient with pruritic erythematous papulopustules resistant to conventional therapies [11]. Close mimics such as dermatomycosis, bacterial folliculitis, rosacea, and mycosis fungoides should be systematically excluded using potassium hydroxide microscopy, culture, and biopsy. An empiric trial of oral indomethacin (25-75 mg/day) may then be used as both a therapeutic and confirmatory strategy when biopsy findings are equivocal. The present case exemplifies this pathway, as the initial biopsy was non-diagnostic, and it was only through persistent clinical suspicion and re-review with follicular sampling that the diagnosis was established. Facial and extrafacial eosinophilic folliculitis have been shown to differ not only clinically but also histopathologically, with perifollicular eosinophilic infiltration, follicular mucinosis, and exocytosis of inflammatory cells into hair follicles being significantly more frequent in facial disease, whereas a perivascular pattern predominates in extrafacial lesions [7]. This site-based histopathologic variation may complicate diagnosis when both facial and extrafacial sites are biopsied simultaneously, as in the present patient. In this patient, the chest biopsy showed a predominantly perivascular eosinophilic pattern without overt folliculotropism, which would not satisfy classic criteria for eosinophilic folliculitis in isolation but is consistent with extrafacial involvement in the appropriate clinical context.

In addition to its variable clinical presentation, the pathophysiology of eosinophilic folliculitis may provide insight into both its diagnostic features and therapeutic response. Prostaglandin-driven inflammation has been proposed as a key contributor to disease activity, with prostaglandin D₂ implicated in eosinophil recruitment and activation within the pilosebaceous unit [12,13]. This mechanistic framework may explain the response to indomethacin, which inhibits cyclooxygenase activity and downstream prostaglandin synthesis. Prior studies have demonstrated that indomethacin can induce rapid and sustained remission in eosinophilic folliculitis, supporting its role as both a therapeutic and diagnostic agent in suspected cases. In the present case, the reproducible response to indomethacin further reinforces this association and highlights its utility in atypical or diagnostically challenging presentations.

## Conclusions

Eosinophilic folliculitis can present with considerable clinical and histopathologic variability, which may delay diagnosis, particularly in patients with persistent pruritic facial eruptions that do not respond to conventional therapies. This case highlights the importance of maintaining a high index of suspicion and emphasizes the role of clinicopathologic correlation in establishing the diagnosis. Given that individual clinical or histologic features may be nonspecific in isolation, integrating clinical presentation with targeted dermatopathologic assessment is essential. In refractory cases, targeted biopsy of follicular structures and consideration of a therapeutic trial of indomethacin may aid in both diagnosis and management. Early recognition may help reduce prolonged morbidity and avoid unnecessary or ineffective treatments. Clinicians should also be aware that eosinophilic folliculitis may occur in the setting of underlying immune dysregulation, warranting appropriate clinical follow-up.
